# Video laryngoscopy does not improve the intubation outcomes in emergency and critical patients – a systematic review and meta-analysis of randomized controlled trials

**DOI:** 10.1186/s13054-017-1885-9

**Published:** 2017-11-24

**Authors:** Jia Jiang, Danxu Ma, Bo Li, Yun Yue, Fushan Xue

**Affiliations:** 1 Department of Anesthesiology, Beijing Chaoyang Hospital, Capital Medical University, Beijing, 100020 China; 2 Beijing Hospital of Traditional Chinese Medicine, affiliated with Capital Medical University, Beijing Institute of Traditional Chinese Medicine, Beijing, 100010 China; 3 Department of Anesthesiology, Plastic Surgery Hospital, Chinese Academy of Medical Sciences and Peking Union Medical College, Beijing, 100144 China

**Keywords:** Airway management, Laryngoscope, Tracheal intubation, Randomized controlled trial

## Abstract

**Background:**

There is significant controversy regarding the influence of video laryngoscopy on the intubation outcomes in emergency and critical patients. This systematic review and meta-analysis was designed to determine whether video laryngoscopy could improve the intubation outcomes in emergency and critical patients.

**Methods:**

We searched the Cochrane Central Register of Controlled Trials, PubMed, Embase, and Scopus databases from database inception until 15 February 2017. Only randomized controlled trials comparing video and direct laryngoscopy for tracheal intubation in emergency department, intensive care unit, and prehospital settings were selected. The primary outcome was the first-attempt success rate. Review Manager 5.3 software was used to perform the pooled analysis and assess the risk of bias for each eligible study. The GRADE (Grading of Recommendations Assessment, Development and Evaluation) system was used to assess the quality of evidence for all outcomes.

**Results:**

Twelve studies (2583 patients) were included in the review for data extraction. Pooled analysis did not show an improved first-attempt success rate using video laryngoscopy (relative risk [RR], 0.93; *P* = 0.28; low-quality evidence). There was significant heterogeneity among studies (*I* ^2^ = 91%). Subgroup analyses showed that, in the prehospital setting, video laryngoscopy decreased the first-attempt success rate (RR, 0.57; *P* < 0.01; high-quality evidence) and overall success rate (RR, 0.58; 95% CI, 0.48–0.69; moderate-quality evidence) by experienced operators, whereas in the in-hospital setting, no significant difference between two devices was identified for the first-attempt success rate (RR, 1.06; *P* = 0.14; moderate-quality evidence), regardless of the experience of the operators or the types of video laryngoscopes used (*P* > 0.05), although a slightly higher overall success rate was shown (RR, 1.11; *P* = 0.03; moderate-quality evidence). There were no differences between devices for other outcomes (*P* > 0.05), except for a lower rate of esophageal intubation (*P* = 0.01) and a higher rate of Cormack and Lehane grade 1 (*P* < 0.01) when using video laryngoscopy.

**Conclusions:**

On the basis of the results of this study, we conclude that, compared with direct laryngoscopy, video laryngoscopy does not improve intubation outcomes in emergency and critical patients. Prehospital intubation is even worsened by use of video laryngoscopy when performed by experienced operators.

**Electronic supplementary material:**

The online version of this article (doi:10.1186/s13054-017-1885-9) contains supplementary material, which is available to authorized users.

## Background

Securing the airway with tracheal intubation (TI) is a fundamental treatment for emergency and critical care patients with respiratory dysfunction or decreased airway protection. Direct laryngoscopy (DL) is the primary method for TI, but it can be challenging when performed in emergencies because the patient often is in life-threatening condition and has the factors that make TI difficult, such as limited mouth opening, unstable cervical spine, blood or secretions in the airway, and facial trauma, and in addition the expertise of available practitioners varies [1, 2]. The first-attempt success rate of urgent TI in emergency and critical patients is relatively low [2–5], and unsuccessful or prolonged TI can be life-threatening and may result in severe complications [6–11].

Video laryngoscopy (VL) is a new device that contains a miniaturized camera at the blade tip to visualize the glottis indirectly. This method was developed at the beginning of the 21st century [12]. It has been shown that VL improves laryngeal visualization compared with DL [13, 14] and provides some advantages in surgical patients, especially those with difficult airways [15–19]. The use of VL in emergent and critical situations has also been tested in several observational studies, which have shown that VL can lead to better intubation outcomes [4, 5, 20, 21]. A recent meta-analysis of intensive care unit (ICU) patients demonstrated that, compared with DL, VL reduces difficult intubation and increases the first-attempt success rate [22]. Of the nine studies included in that meta-analysis, however, only three (*n* = 157 subjects) were randomized controlled trials (RCTs) [23–25]. An observational study, whether prospective, nonrandomized, or retrospective in design, does not control for the operators’ experience with each device or for patients’ conditions and thus may bias the determination of the efficacy of different airway devices.

Recently, the performance of VL and DL in patients needing emergency TI was compared in several RCTs, and some of them showed no benefit regarding success rate or intubation time with VL [24, 26–32]. In view of this, we performed a systematic review and meta-analysis that included only RCTs comparing the performance of VL and DL for emergency TI with respect to the intubation outcomes and complications. Our review is registered with PROSPERO (http://www.crd.york.ac.uk/PROSPERO, CRD42017054804).

## Methods

### Data sources

The Cochrane Central Register of Controlled Trials (CENTRAL), PubMed, Embase, and Scopus databases were searched from inception of the databases until 15 February 2017. The PubMed search strategy provided in Additional file 1 was applied to search other electronic databases. For literature without full text, the corresponding author of the study was contacted by email. The reference lists of all eligible trials and reviews were screened for additional citations. No language restriction was imposed.

### Study selection

RCTs or quasi-RCTs comparing VL and DL for TI in emergency or critical care patients were included. Manikin studies, cadaveric studies, and retrospective or observational studies were excluded. Participants were nonsurgical patients needing emergent TI in the in-hospital or prehospital setting. Patients with suspected laryngeal trauma or extensive maxillofacial injury requiring an immediate surgical airway, supraglottic airway, or awake fiberoptic intubation were excluded. The primary outcome was the first-attempt success rate. The secondary outcomes were overall success rate; duration of intubation; and complications, including esophageal intubation, aspiration, severe low oxygen saturation, and in-hospital mortality. The rate of Cormack and Lehane grade 1 classification was also recorded. The definitions of the outcomes are shown in Additional file 2: Table S1.

The titles and abstracts were independently screened by two of the present review’s authors (JJ and DM). After retrieving the full texts of any potentially relevant studies, the studies’ eligibility was determined. Any disagreements between the two review authors were resolved by discussion with the other authors until a consensus was obtained. A Preferred Reporting Items for Systematic Review and Meta-Analysis (PRISMA) flow diagram was completed to record the selection process in sufficient detail [33].

### Data extraction and risk of bias assessment

The data were independently extracted by two review authors (JJ and DM). For continuous data, mean, SD, and sample size were extracted. Data such as median or CI that cannot be used directly were converted to SD by using a formula provided in the Cochrane Handbook [34]. For dichotomous variables, the number of events that had occurred and the sample size were extracted. The corresponding author of the study was contacted if the data were unavailable.

The risk of bias for each eligible study was independently assessed by two review authors (JJ and DM) using the “risk of bias” assessment tool of the Cochrane Handbook [34]. If all seven domains were assigned a low risk of bias, the study was classified as “low risk”; if one or more domains were assigned to the “unclear risk” of bias category, the study was classified as “unclear risk”; if one or more domains were assigned to a high risk of bias, the study was classified as “high risk” [34]. Furthermore, the criteria of the Grading of Recommendations Assessment, Development and Evaluation (GRADE) system (study limitations, consistency of effect, imprecision, indirectness, and publication bias) were used to assess the quality of the body of evidence associated with all outcomes [35, 36]. Then we developed a grade evidence profile table using the GRADE software (www.guidelinedevelopment.org) to rate these outcomes as being of high, moderate, low, or very low quality. If serious or very serious deficiencies in these criteria were considered, the quality of evidence was downgraded by one or two levels.

### Data analysis

The weighted mean difference (WMD) and 95% CI were used for continuous data. Relative risk (RR) and 95% CI were used for dichotomous data. A *P* value less than 0.05 was considered statistically significant. RevMan 5.3 software (Cochrane Collaboration, London, UK) was used to perform the pooled analysis for the outcomes from more than one study. A chi-square test with the *I* ^2^ statistic was used to quantify heterogeneity. An *I* ^2^ value less than 40% was considered as low heterogeneity, and a fixed-effect model was used; otherwise, a random-effect model was used. In the presence of statistical heterogeneity (*I* ^2^ ≥ 40%) or an indication of clinical heterogeneity, subgroup analysis was planned for the primary outcomes according to the following possible heterogeneous factors: (a) different settings: in-hospital (ICU and emergency room) and prehospital; (b) operators’ experience: experienced (certified anesthesiologist, emergency medical service technician with more than 3 years of clinical experience, performed > 50 TIs, or according to the judgment of the study authors) or inexperienced; and (c) different devices: channeled, Macintosh and angulated VLs. Sensitivity analysis was conducted to explore other potential sources of heterogeneity if necessary. Reporting bias was assessed using funnel plots if the result of the primary outcome was derived from at least ten trials [37].

## Results

**Fig. 1:**
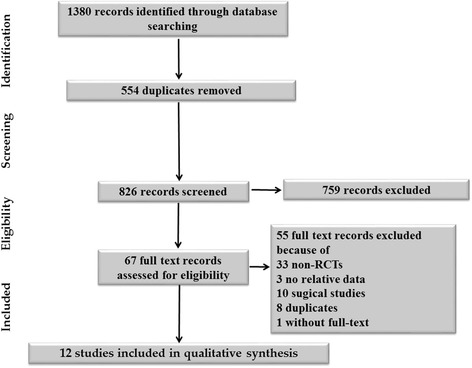
Search process for identified records. *RCT* Randomized controlled trial

Using our search strategy, a total of 1380 papers were identified. Of them, 1313 were excluded during title and abstract screening because they were duplicates or irrelevant to our research question. Sixty-seven studies were selected for full-text assessment using our inclusion and exclusion criteria. Fifty-two further studies were removed because of having non-RCT characteristics, lacking relevant data, including surgical participants, and/or being duplicates. Authors of three studies were contacted for their full-text articles to confirm their eligibility: One [38] proved to be a meeting report that was part of another included study [24]; one was an observational study [39]; and another was qualified, but its full text could not be obtained to do the risk of bias assessment [40]. Thus, all three of these studies were excluded. One study author was contacted for additional data [41]. Eventually, 12 studies (*n* = 2583) were included in the review for data extraction [23–32, 41, 42]. The study selection process is shown in Fig. 1.

### Description of included studies

Characteristics of included studies are listed in Table 1. Among 12 included studies, 10 were RCTs, and the remainder were quasi-RCTs; 3 were carried out in the prehospital setting and 9 in the ICU or emergency department (in-hospital setting). Most intubations in seven studies were performed by experienced operators, and five were performed mostly by inexperienced operators. The angulated VL (GlideScope; Verathon, Seattle, WA, USA) was used in six studies, the Macintosh-type VL in four (C-MAC, Karl Storz, Tuttlingen, Germany; or McGrath MAC, Aircraft Medical, Edinburgh, Scotland), and the channeled VL in two (Pentax Airway Scope, Pentax Lifecare/Hoya, Tokyo, Japan; or Airtraq, Prodol Meditec, Las Arenas, Spain). Five in-hospital studies excluded patients with cardiac arrest, and one enrolled only patients with cardiac arrest. Rapid sequence induction (RSI) with sedatives or narcotics and neuromuscular blockades (NMBAs) were chosen for all participants or as appropriate by choice of physicians in most included studies. Three studies did not use any NMBAs [24, 26, 29].

**Table 1:** Characteristics of the 12 included studies

First author, year [reference]	Design	Settings	No. of patients	Participants	Difficult airways included	Devices	Operators (experience)	RSI	NMBAs
Arima et al., 2014 [26]	Quasi-RCT	Prehospital	109	Age ≥ 18 years	No	Airway Scope vs. DL	Physicians (experienced)	None	None
Driver et al., 2016 [42]	RCT	ED	198	Adult patients	No	C-MAC vs. DL	Senior ED residents (most experienced)	Most	Most
Goksu et al., 2016 [27]	RCT	ED	150	Age ≥ 16 years	No	C-MAC vs. DL	ED residents and attending physicians (most inexperienced)	All	All^a^
Griesdale et al., 2012 [23]	RCT	ICU	40	Age ≥ 16 years without cardiac arrest	Yes	GlideScope vs. DL	Novice providers (inexperienced)	All	All
Janz et al., 2016 [28]	RCT	ICU	150	Age ≥ 18 years	No	McGrath MAC (98.6%), GlideScope (1.4%) vs. DL	Trained pulmonary and critical care medicine fellows (inexperienced)	All	Most
Kim et al., 2016 [29]	RCT	ED	140	Adult patients with CPR	No	GlideScope vs. DL	Experienced intubators (>50 successful TIs)	None	None
Lascarrou et al., 2017 [30]	RCT	ICU	371	Adult patients without CPR	Yes	McGrath MAC vs. DL	311 inexperienced (84.8%) and 60 experienced intubators (most inexperienced)	All	All
Silverberg et al., 2015 [24]	Quasi-RCT	ICU	117	SpO_2_ < 92% after mask ventilation excluded	Yes	GlideScope vs. DL	Trained pulmonary and critical care medicine fellows (inexperienced)	As needed	None
Sulser et al., 2016 [31]	RCT	ED	150	Adult patients without CPR	No	C-MAC vs. DL	Anesthesia consultants (experienced)	As needed	All
Trimmel et al., 2011 [41]	RCT	Prehospital	212	Adult patients	No	Airtraq vs. DL	Anesthesiologists or EMS physicians (experienced)	As needed	As needed
Trimmel et al., 2016 [32]	RCT	Prehospital	326	Adult patients	No	GlideScope vs. DL	EMS physicians (experienced)	As needed	As needed
Yeatts et al., 2013 [25]	RCT	ED	623	CPR patients excluded	No	GlideScope vs. DL	ED or anesthesiology residents (experienced)	All	All

*Abbreviations: RSI* Rapid sequence induction, *NMBAs* Neuromuscular blockades, *RCT* Randomized controlled trial, *ED* Emergency department, *ICU* Intensive care unit, *CPR* Cardiopulmonary resuscitation, *DL* Direct laryngoscopy, *TI* Tracheal intubation, *EMS* Emergency medical service, *SpO*_*2*_ Oxygen saturation by pulse oximetry

^a^The author was contacted to confirm this issue

**Fig. 2:**
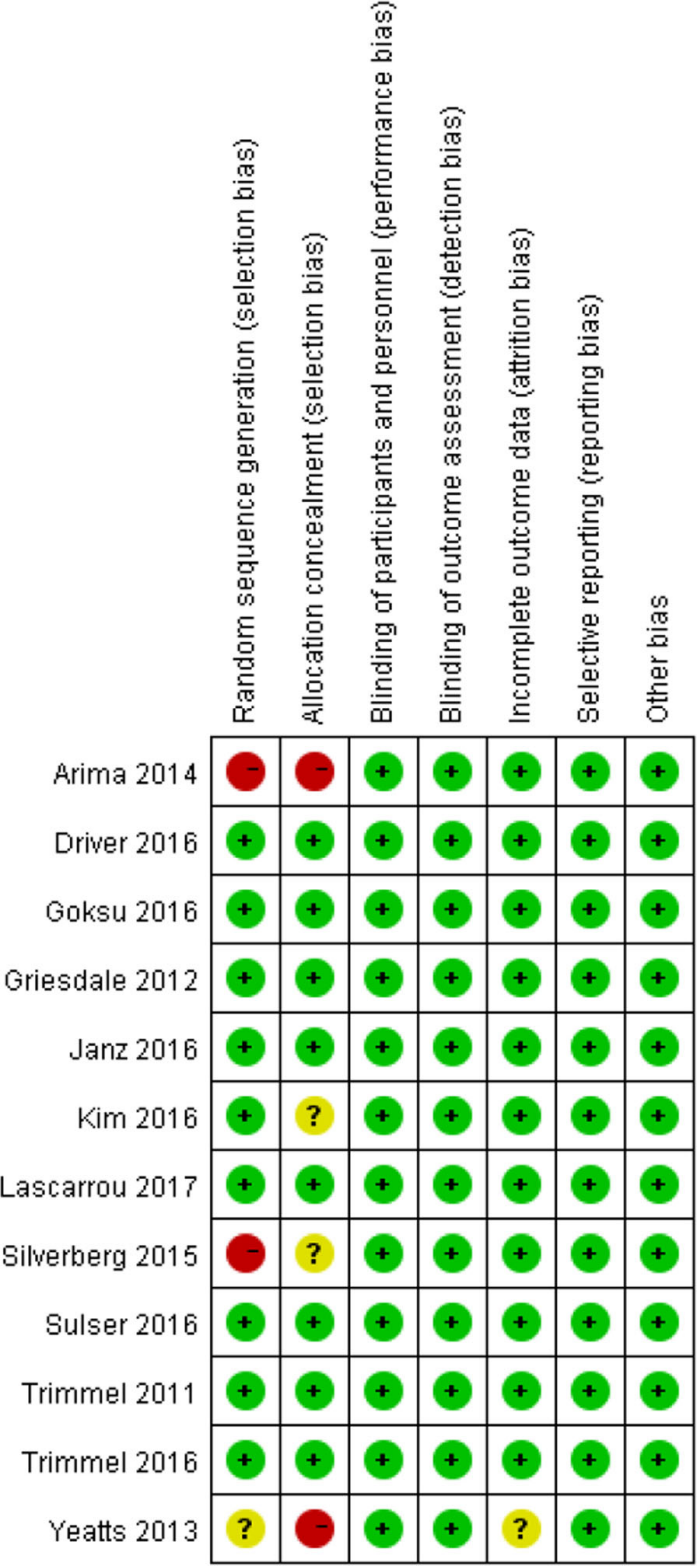
Risk of bias summary: judgments about each risk of bias item for each included study based on quality evaluation of 12 included studies. + Low risk, − High risk, ? Unknown

The overall risk of bias of the included studies was relatively low. Eight of them could be classified as low-risk studies and three as high-risk studies. Detailed information regarding the risk of bias of the included studies is shown Fig. 2 and summarized in Additional file 3: Table S2. A funnel plot obtained from the primary outcome is shown in Additional file 4: Figure S1. The GRADE system showed that the quality of most evidence was low or moderate for inconsistency owing to a moderate or high level of heterogeneity. The results of the evidence of outcomes are listed in Additional file 5: Table S3.

### First-attempt success rate

The data on the first-attempt success rate for all 12 included studies were available. Pooled analysis showed no significant difference in the first-attempt success rate between VL and DL (12 studies; RR, 0.93; 95% CI, 0.82–1.06; *n* = 2583; *P* = 0.28; low-quality evidence). There was significant heterogeneity among studies (*P* < 0.01; *I* ^2^ = 91%) (Additional file 6: Figure S2).

**Fig. 3:**
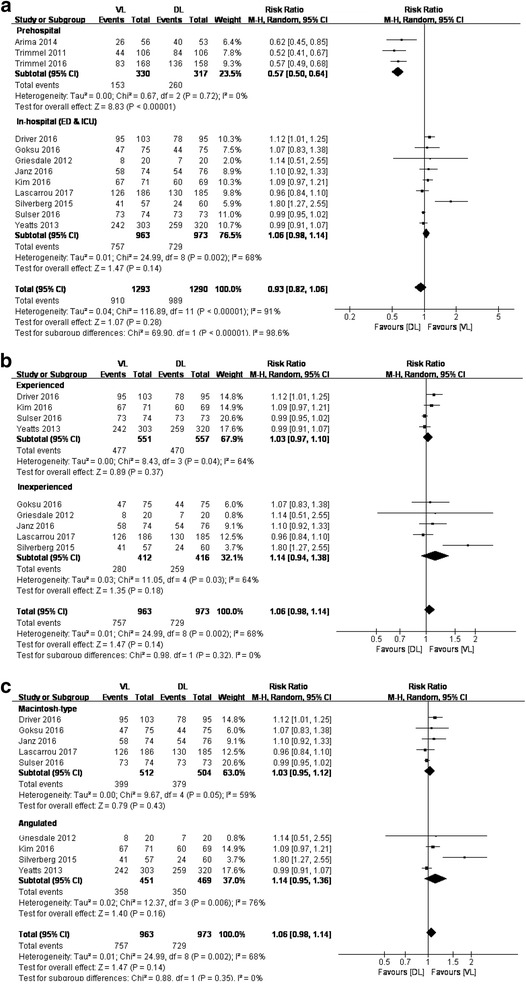
VL vs. DL for first-attempt success rate based on different settings (**a**), experience levels of operators in in-hospital settings (**b**), and different types of video laryngoscopy in in-hospital settings (**c**). *VL* Video laryngoscopy, *DL* Direct laryngoscopy, *M-H* Mantel-Haenszel

Subgroup analysis according to different settings identified a significant difference for the prehospital setting (three studies; RR, 0.57; *n* = 647; *P* < 0.01; high-quality evidence) but no significant difference for the in-hospital setting (nine studies; RR, 1.06; *n* = 1936; *P* = 0.14; moderate-quality evidence). Prehospital intubation was performed mostly by experienced operators, and two kinds of VLs (channeled [26, 41] or angulated [32] VLs) were used. Given that different settings would introduce principal heterogeneity and only three studies in the prehospital setting were included, subgroup analyses based on the experience of operators and different devices used were performed only in the in-hospital setting. No significant difference was found when TI was performed by experienced operators (four studies; RR, 1.03; *n* = 1108; *P* = 0.37) or by inexperienced operators (six studies with seven comparisons; RR, 1.16; *n* = 924; *P* = 0.05). No significant difference was found for intubation with angulated VLs (GlideScope; five studies with six comparisons; RR, 1.16; *n* = 1016; *P* = 0.04) or with Macintosh-type VLs (C-MAC/McGrath MAC; five studies; RR, 1.03; *n* = 1016; *P* = 0.43) (Fig. 3). The study by Silverberg et al. [24] had a much higher first-attempt success rate when using VL than that in other studies, and it was the only study performed in non-cardiopulmonary resuscitation (non-CPR) patients without using any NMBAs. Thus, a sensitivity analysis excluding this study in the in-hospital setting was conducted. The results were not altered; however, no evidence of heterogeneity could be found in all subgroups that originally included this study (*I* ^2^ < 40%).

### Secondary outcomes

Results of secondary outcomes, including overall success rate, duration of intubation, esophageal intubation rate, in-hospital mortality, aspiration, severe low oxygen saturation, and Cormack and Lehane grade 1 classification, are summarized in Table 2 and Additional file 7: Figure S3, S4, S5, and S6.

**Table 2:** Results of meta-analysis for secondary outcomes between direct laryngoscopy and video laryngoscopy

Outcomes	Studies (*n*)	Participants (*n*)	Heterogeneity	Heterogeneity statistical method	Effect estimate (*P* value)
Overall success rate	8	1292	*P* < 0.01; *I* ^2^ = 97%	Risk ratio (M-H, random, 95% CI)	0.86 [0.67–1.09] (*P* < 0.01)
Overall success rate (prehospital)	3	647	*P* = 0.04; *I* ^2^ = 69%	Risk ratio (M-H, random, 95% CI)	0.58 [0.48–0.69] (*P* < 0.01)
Overall success rate (in-hospital)	5	645	*P* = 0.008; *I* ^2^ = 71%	Risk ratio (M-H, random, 95% CI)	0.86 [0.67–1.09] (*P* = 0.03)
Duration of intubation	10	2173	*P* < 0.01; *I* ^2^ = 88%	WMD (IV, random, 95% CI)	-2.12[-13.41-9.18] (*P* = 0.71)
Esophageal intubation rate	6	1245	*P* = 0.72; *I* ^2^ = 0%	Risk ratio (nonevent) (M-H, random, 95% CI)	0.36 [0.16–0.80] (*P* = 0.01)
In-hospital mortality	6	1494	*P* = 0.89; *I* ^2^ = 0%	Risk ratio (nonevent) (M-H, random, 95% CI)	1.12 [0.86–1.45] (*P* = 0.39)
Aspiration rate	6	1588	*P* = 0.007; *I* ^2^ = 69%	Risk ratio (nonevent) (M-H, random, 95% CI)	1.01 [0.98–1.03] (*P* = 0.52)
Severe low oxygen saturation rate	4	664	*P* = 0.07; *I* ^2^ = 57%	Risk ratio (M-H, random, 95% CI)	1.43 [0.51–3.96] (*P* = 0.50)
Proportion of C&L grade 1 classification	4	690	*P* < 0.01; *I* ^2^ = 20%	Risk ratio (M-H, fixed, 95% CI)	1.54 [1.37–1.74] (*P* < 0.01)

*Abbreviations: C&L* Cormack and Lehane, *IV* Inverse variation, *M-H* Mantel-Haenszel, *WMD* Weighted mean difference

## Discussion

To our knowledge, this is the first meta-analysis and systematic review of available RCTs comparing VL and DL for TI in emergency and critical care patients, including the quality of evidence. In this analysis, the first-attempt success rate was used as the primary endpoint because multiple intubation attempts performed outside the operating room can significantly increase the risk of life-threatening complications [6, 43, 44]. Furthermore, improving the first-attempt success rate has been regarded as the main goal of emergency TI [45]. Our results show that laryngeal visualization was improved by using VL. This is consistent with findings for surgical patients in the operating room [12]. However, better visualization did not translate into an improved first-attempt success rate or other intubation outcomes or complications, except for a lower rate of esophageal intubation. Prehospital intubation outcomes were even worsened with lower first-attempt and overall success rates with VL when TI was performed by experienced operators.

Evidence derived from surgical patients shows that VL is associated with better intubation outcomes, especially for inexperienced operators and patients with difficult airways [16, 28, 46]. This is because TI in the operating room is controllable, such as with the common use of RSI and NMBAs, patients’ fasting state, and favorable oxygenation, as well as appropriate light or intubation position. For highly experienced anesthesiologists, it seems unlikely that a single device will show superiority unless a difficult airway is encountered [47–51], whereas for novices who have not yet received long-term DL training, visualization of the airway on a video screen can allow their supervisors to directly assist them in completing an intubation themselves, thus reducing the number of attempts and improving the safety of airway management [52]. However, emergent TI is quite another thing. Although TI in the emergency department or ICU is frequently performed by paramedics or emergency medicine physicians who do not practice TI with DL on a daily basis [53], and although the patients often have a higher risk of difficult airways [9, 54], the operators may not benefit from using VL as novices in the operating room. There are several uncontrollable factors that may explain this difference. First, critically ill patients with a poor oxygen reserve capacity are more subject to hypoxia, which makes it more likely that operators will turn to alternatives such as DL, a flexible or rigid bronchoscope, or at least further mask oxygenation. If TI is not completed within the allowed time, inexperienced operators will be replaced by more experienced operators earlier, making the first-attempt success rate much lower. Second, secretions or blood in the airway might impair laryngeal visualization with VLs [26, 28]. Third, RSI and NMBAs will be chosen with caution owing to circulation compromise, certain airway problems, operators’ experience, or accessibility of medicine. Prehospital intubation is more challenging, owing to additional risk factors such as ambient light, limited workspace, special positioning, and chest compression during CPR [55]. Under chest compression, increased intrathoracic pressure can cause reflux of gastric contents, resulting in more attempts and longer intubation time with the VL. Prolonged intubation time and subsequent hypoxemia have been identified as major reasons for increased mortality in patients undergoing prehospital intubation [56]. In addition, in prehospital care, DL is more accessible, and most operators are experienced in using it.

It must be emphasized that performance of VL is different between devices owing to various designs and shapes [57, 58]. Even a slight design modification may significantly change the success rate, intubation time, and use of adjunct maneuvers [59]. Some types of VLs have their own design-related deficiencies that may dwarf their results. For example, the A.P. Advance™ VL (Venner Medical International, St Helier, Jersey, UK), with a large video screen, shows the plastic part of the blade tip instead of the relevant airway, contributing to its poor performance [58]. Studies included in our analysis used three types of VLs (angulated, Macintosh, or channeled), including five different devices (GlideScope, C-MAC, McGrath MAC, Airwayscope, and Airtraq). In the prehospital setting, two of three included studies used channeled VL. The channeled VL, with its integrated design, might be more portable in the prehospital setting, but it is bulkier and may require other team members to maneuver the tracheal tube [58]. It should be noted that the poor performance of the VL is due mainly to the prehospital setting itself rather than to the devices chosen. We therefore did a related subgroup analysis only in the in-hospital setting. No difference was identified between VLs and DLs, regardless of the devices used. Although an angled blade design was assumed to facilitate laryngeal visualization and thus to lead to a better intubation outcome, it may afford less room for tracheal tube insertion and increase stylet use in patients with a normal airway, resulting in increased procedural difficulty and prolonged intubation time [25, 60]. In addition, pooling of results from studies evaluating different VLs may lead to intrinsic inconsistencies. An especially important issue neglected in the design of the five included studies comparing the Macintosh-type VL and DL is that the Macintosh-type VL can provide the two options of DL and VL in one device. When one attempt fails, the operators can immediately switch to another option to successfully complete the TI without having to make a second attempt [61]. This unique feature of Macintosh-type VLs is significantly different from DLs and angulated VLs, which can provide only one option. Thus, definition of laryngoscopy attempts used in these studies is desirable for DLs but not for Macintosh-type VLs [62].

The results of some studies indicate that VLs should be used with caution in critical patients because of a prolonged intubation time and subsequent possible higher incidence of severe life-threatening complications [23, 25, 30]. Our review shows that incidences of aspiration, severe low oxygen saturation, and in-hospital deaths did not differ between VLs and DLs. However, these results remain unreliable owing to the limited number of participants included. Our review shows a lower rate of esophageal intubation using VLs than that in another study [22]. This might be somewhat meaningful because “even a single episode of recognized esophageal intubation is associated with desaturation, increased risk of aspiration, and cardiac arrest” [63]. Moreover, an important and promising finding in one of our included studies and another observational study is that the use of a VL has a higher first-attempt success rate with fewer chest compression interruptions in the emergency department [29, 64].

Our study included only RCTs and quasi-RCTs. Although blinding was not adopted in most studies, we judged “no blinding” as low risk because it seems impossible to blind personnel in urgent situations at times. In the prehospital setting, moreover, there is never time for allocation concealment, and even randomization using a common method such as a random number table is impractical. Risk assessment of bias for the included studies showed that 7 of 12 studies could be classified as low-risk studies. Therefore, in general, this supports the quality of our study. The funnel plot, with its visually symmetrical distribution, qualitatively indicates a low risk of publication bias. Given that the quality of most evidence was low or moderate owing to a moderate or high level of heterogeneity, subgroup analysis and sensitivity analysis based on some potential clinical heterogeneous factors also were performed in our review.

There are some limitations of our review. First, although subgroup analyses were performed, there were still other clinical heterogeneities in subgroups, such as patients having different conditions, use of various intubation strategies, and use of any adjacent tool or maneuver. Whether patients with predicted difficult airways were enrolled was another important heterogeneous factor. However, for emergency or critical care patients, the traditional predictors of difficult airways, such as thyromental distance, Mallampati score, or neck mobility, cannot be recorded, because all intubations are performed so urgently that there is never a chance to make predictions or subsequent grouping before randomization. One observational study showed that VLs significantly increased the intubation success rate in emergency patients with difficult airways [65]. In the absence of a difficult airway, however, the use of VLs may even bring some disadvantages [25]. Whether anesthetics were used and the choice of medication can also introduce heterogeneity. RSI with sedatives, narcotics, and NMBAs has been shown to facilitate TI and decrease intubation-related complications in reasonable circumstances [66, 67]. Because most of the studies included in our review did not have strict protocols regarding medication, subgroup analysis according to medications seemed impossible. Anyway, the study by Silverberg et al. [24] demonstrated a much higher first-attempt success rate using VLs. Sensitivity analysis excluding this study did not alter the results, but the heterogeneities within the subgroups disappeared, indicating that this study may be the main factor leading to heterogeneity. The effect of the NMBAs on the result was unclear. It may be the negative influence of alternating of devices that use different configurations on the learning curve of operators with the DLs that led to a lower success rate with DLs. Second, owing to ethical considerations, some patients had to be excluded on enrollment, such as patients with low oxygen saturation [24], those with an immobilized cervical spine, and patients with predicted difficult airways, or those excluded owing to attending physicians’ discretion and unavailability of devices at the time of eligible patient arrival [25]. It is unclear whether these excluded patients would benefit from one of the interventions. Third, the classification of the operators’ qualifications and the definition of intubation time or overall success rate used in our analysis were based on previous papers or our own judgment, and this might somehow be arbitrary.

## Conclusions

This review does not reveal any improvements in intubation outcomes with the use of VLs compared with DLs in emergency and critical care patients, except for a lower rate of esophageal intubation with VLs. In the prehospital setting, intubation outcomes may be worsened by the VL when intubation is performed by experienced operators. Further studies are still needed to determine whether the VL is beneficial for emergency and critical care patients with difficult airways, regardless of the operator’s experience, and should be focused more on the impact of VLs on prognostic outcomes such as severe complications, length of hospital stay, and mortality.

## Additional files

Additional file 1: The PubMed search strategy. (DOC 23 kb)

Additional file 2: Table S1. Definitions of some outcomes. (DOC 28 kb)

Additional file 3: Table S2. Description of the risk of bias for 12 included studies. (DOC 88 kb)

Additional file 4: Figure S1. Funnel plot of comparison for the primary outcome: first-attempt success rate. (DOC 32 kb)

Additional file 5: Table S3. GRADE evidence profile of all outcomes. (DOC 94 kb)

Additional file 6: Figure S2. VL vs. DL for first-attempt success rate. *Abbreviations: VL* Video laryngoscope, *DL* Direct laryngoscope. (DOC 35 kb)

Additional file 7: **Figures S3**, **S4**, **S5**, and **S6** VL vs. DL for overall success rate. *Abbreviations: VL* Video laryngoscope, *DL* Direct laryngoscope. (DOC 97 kb)

## Abbreviations

C&L: Cormack and Lehane
CENTRAL: Cochrane Central Register of Controlled Trials
CPR: Cardiopulmonary resuscitation
DL: Direct laryngoscopy
ED: Emergency department
EMS: Emergency medical service
GRADE: Grading of Recommendations Assessment, Development and Evaluation
ICU: Intensive care unit
IV: Inverse variation
M-H: Mantel-Haenszel
NMBA: Neuromuscular blockade
PRISMA: Preferred Reporting Items for Systematic Review and Meta-Analysis
RCT: Randomized controlled trial
RR: Relative risk
RSI: Rapid sequence induction
SpO_2_: Oxygen saturation by pulse oximetry
TI: Tracheal intubation
VL: Video laryngoscopy
WMD: Weighted mean difference
