# Supplementary material for: Video laryngoscopy does not improve the intubation outcomes in emergency and critical patients – a systematic review and meta-analysis of randomized controlled trials
Source: Crit Care. 2017 Nov 24;21:288. doi: 10.1186/s13054-017-1885-9 (PMC5702235; doi:10.1186/s13054-017-1885-9)
Supplement: Supplementary file 1 — The PubMed search strategy. (DOC 23 kb) [file 13054_2017_1885_MOESM1_ESM.doc]

**Additional file 1**

(((((((((((((((((BERCI[Title/Abstract]) OR "Storz DCI"[Title/Abstract]) OR "Venner APA") OR "TruView PCD"[Title/Abstract]) OR "Pentax AWS"[Title/Abstract]) OR "Airway Scope"[Title/Abstract]) OR Airtraq[Title/Abstract]) OR C-MAC[Title/Abstract]) OR Glidescope[Title/Abstract]) OR McGrath[Title/Abstract]) OR "King Vision"[Title/Abstract]) OR ((((videolaryngoscope[Title/Abstract]) OR "airway scope"[Title/Abstract]) OR video laryngoscopy[Title/Abstract]) OR video laryngoscope[Title/Abstract]))) AND (((((((((((groups[Title/Abstract])) OR (trial[Title/Abstract])) OR (randomly[Title/Abstract])) OR (drug therapy[MeSH Subheading])) OR (placebo[Title/Abstract])) OR (randomized[Title/Abstract])) OR (controlled clinical trial[Publication Type])) OR ("Randomized Controlled Trial"[Publication Type]))) NOT (((animals[MeSH Terms])) NOT (((animals[MeSH Terms])) AND (humans[MeSH Terms])))))) NOT (((((("simulation study"[Title/Abstract]) OR "retrospective study"[Title/Abstract]) OR "observational study"[Title/Abstract]) OR cadaver[Title/Abstract]) OR mannequin[Title/Abstract]) OR manikin[Title/Abstract]))) NOT ((((neonate [Title/Abstract]) OR infant [Title/Abstract]) OR pediatric [Title/Abstract]) OR children [Title/Abstract])
