# Supplementary material for: Video laryngoscopy does not improve the intubation outcomes in emergency and critical patients – a systematic review and meta-analysis of randomized controlled trials
Source: Crit Care. 2017 Nov 24;21:288. doi: 10.1186/s13054-017-1885-9 (PMC5702235; doi:10.1186/s13054-017-1885-9)
Supplement: Supplementary file 2 — Definitions of some outcomes. (DOC 28 kb) [file 13054_2017_1885_MOESM2_ESM.doc]

**Additional file 2: Table S1. Definitions of some outcomes.**

| **Outcome** | **Definition** |
| --- | --- |
| First-attempt success rate | The proportion of successful placement of a tracheal tube in the trachea during first insertion of a laryngoscope into oral cavity without removing the device from the mouth. Suction and stylets could be used. |
| Overall successful intubation rate | The final proportion of successful intubation performed with the preferred device regardless of the number of attempts, and only data from the studies which supposed not to switch to another intubation method just after a single failed attempt would be selected. |
| Duration of intubation | The time from the first attempt at insertion of the laryngoscope to the confirmation of the tube placement in the trachea by using study’s own method (such as auscultation, fogging in the tube, or P_ET_CO_2_) |
| Aspiration | Emesis during intubation or witnessed oral content into trachea or aspiration pneumonia was identified. |
| Severe low oxygen saturation | Oxygen saturation < 80% |
| In-hospital mortality | Follow-up for 28 days |
