# Supplementary material for: Video laryngoscopy does not improve the intubation outcomes in emergency and critical patients – a systematic review and meta-analysis of randomized controlled trials
Source: Crit Care. 2017 Nov 24;21:288. doi: 10.1186/s13054-017-1885-9 (PMC5702235; doi:10.1186/s13054-017-1885-9)
Supplement: Supplementary file 3 — Description of the risk of bias for 12 included studies. (DOC 88 kb) [file 13054_2017_1885_MOESM3_ESM.doc]

**Additional file 4: S2. Description of the Risk of Bias for 12 Included Studies.**

| **Domains** | **Description** |
| --- | --- |
| Random sequence generation | Nine studies [23, 27-32, 41, 42] clearly described the methods for the generation of randomized sequences, one study [25] had unclear methods for the generation of randomized sequences (The authors have been contacted for detailed method of randomization, however, the email address was invalid.), and the other two [24, 26] were quasi randomized trial. |
| Allocation concealment | Eight studies [23, 27, 28, 30-32, 41, 42] used nontransparent envelopes or other method to conceal the allocation, two [25, 26] did not conceal the allocation, and the other two [24, 29] did not mention whether allocation concealment was used. |
| Blinding of participants, personnel, and outcome assessment | Although no study used blinded method, the authors judged that the outcome would not be likely to be influenced as the patients were under emergent setting and not aware of their grouping. Moreover, it is always impossible to blind the personnel due to the nature of the setting and intervention. |
| Incomplete outcome data | One study lost 24.2% data on intubation outcomes and did not provide reasons for missing data [25]. Two studies excluded patients with balanced numbers across two groups and similar reasons for missing data [26, 31]. No missing data was reported in other studies. |
| Selective reporting | The published studies reported all expected outcomes. |
| Other bias | The study appeared to be free of other sources of bias. |
