# Supplementary material for: Video laryngoscopy does not improve the intubation outcomes in emergency and critical patients – a systematic review and meta-analysis of randomized controlled trials
Source: Crit Care. 2017 Nov 24;21:288. doi: 10.1186/s13054-017-1885-9 (PMC5702235; doi:10.1186/s13054-017-1885-9)
Supplement: Supplementary file 5 — GRADE evidence profile of all outcomes. (DOC 94 kb) [file 13054_2017_1885_MOESM5_ESM.doc]

**Additional file 5: Table S3. Grade Evidence Profile of all Outcomes**

| **Quality assessment** | | | | | | | **No of patients** | | **Effect** | | **Quality** | **Importance** |
| --- | --- | --- | --- | --- | --- | --- | --- | --- | --- | --- | --- | --- |
|  |  |  |  |  |  |  |  |  |  |  |  |  |
| **No of studies** | **Design** | **Risk of bias** | **Inconsistency** | **Indirectness** | **Imprecision** | **Other considerations** | **Video laryngoscope** | **Direct laryngoscope** | **Relative (95% CI)** | **Absolute** |  |  |
| **First attempt success rate** | | | | | | | | | | | | |
| 12 | randomised trials | no serious risk of bias | very serious^1^ | no serious indirectness | no serious imprecision | none | 910/1293  (70.4%) | 989/1290  (76.7%) | RR 0.93 (0.82 to 1.06) | 54 fewer per 1000 (from 138 fewer to 46 more) | ⊕⊕OO LOW | CRITICAL |
|  |  |  |  |  |  |  |  | 77.4% |  | 54 fewer per 1000 (from 139 fewer to 46 more) |  |  |
| **First attempt success rate - Prehospital** | | | | | | | | | | | | |
| 3 | randomised trials | no serious risk of bias | no serious inconsistency | no serious indirectness | no serious imprecision | none | 153/330  (46.4%) | 260/317  (82%) | RR 0.57 (0.5 to 0.64) | 353 fewer per 1000 (from 295 fewer to 410 fewer) | ⊕⊕⊕⊕ HIGH | CRITICAL |
|  |  |  |  |  |  |  |  | 79.3% |  | 341 fewer per 1000 (from 285 fewer to 397 fewer) |  |  |
| **First attempt success rate - In-hospital (ED & ICU)** | | | | | | | | | | | | |
| 9 | randomised trials | no serious risk of bias | serious | no serious indirectness | no serious imprecision | none | 757/963  (78.6%) | 729/973  (74.9%) | RR 1.06 (0.98 to 1.14) | 45 more per 1000 (from 15 fewer to 105 more) | ⊕⊕⊕O MODERATE | CRITICAL |
|  |  |  |  |  |  |  |  | 71.1% |  | 43 more per 1000 (from 14 fewer to 100 more) |  |  |
| **First attempt success rate (in-hospital)- Experienced** | | | | | | | | | | | | |
| 4 | randomised trials | no serious risk of bias | serious^2^ | no serious indirectness | no serious imprecision | none | 477/551  (86.6%) | 470/557  (84.4%) | RR 1.03 (0.97 to 1.1) | 25 more per 1000 (from 25 fewer to 84 more) | ⊕⊕⊕O MODERATE | CRITICAL |
|  |  |  |  |  |  |  |  | 84.5% |  | 25 more per 1000 (from 25 fewer to 85 more) |  |  |
| **First attempt success rate (in-hospital) - Inexperienced** | | | | | | | | | | | | |
| 5 | randomised trials | no serious risk of bias | serious^2^ | no serious indirectness | no serious imprecision | none | 280/412  (68%) | 259/416  (62.3%) | RR 1.14 (0.94 to 1.38) | 87 more per 1000 (from 37 fewer to 237 more) | ⊕⊕⊕O MODERATE | CRITICAL |
|  |  |  |  |  |  |  |  | 58.7% |  | 82 more per 1000 (from 35 fewer to 223 more) |  |  |
| **First attempt success rate (in-hospital)- Macintosh-type** | | | | | | | | | | | | |
| 5 | randomised trials | no serious risk of bias | serious^2^ | no serious indirectness | no serious imprecision | none | 399/512  (77.9%) | 379/504  (75.2%) | RR 1.03 (0.95 to 1.12) | 23 more per 1000 (from 38 fewer to 90 more) | ⊕⊕⊕O MODERATE | CRITICAL |
|  |  |  |  |  |  |  |  | 71.1% |  | 21 more per 1000 (from 36 fewer to 85 more) |  |  |
| **First attempt success rate (in-hospital)- Angulated** | | | | | | | | | | | | |
| 4 | randomised trials | no serious risk of bias | serious^2^ | no serious indirectness | no serious imprecision | none | 358/451  (79.4%) | 350/469  (74.6%) | RR 1.14 (0.95 to 1.36) | 104 more per 1000 (from 37 fewer to 269 more) | ⊕⊕⊕O MODERATE | CRITICAL |
|  |  |  |  |  |  |  |  | 60.5% |  | 85 more per 1000 (from 30 fewer to 218 more) |  |  |
| **Overall successful intubation** | | | | | | | | | | | | |
| 8 | randomised trials | no serious risk of bias | very serious^1^ | no serious indirectness | no serious imprecision | none | 501/655  (76.5%) | 584/637  (91.7%) | RR 0.86 (0.67 to 1.09) | 128 fewer per 1000 (from 303 fewer to 83 more) | ⊕⊕OO LOW | IMPORTANT |
|  |  |  |  |  |  |  |  | 91.4% |  | 128 fewer per 1000 (from 302 fewer to 82 more) |  |  |
| **Overall successful intubation - Prehospital** | | | | | | | | | | | | |
| 3 | randomised trials | no serious risk of bias | serious^2^ | no serious indirectness | no serious imprecision | none | 188/330  (57%) | 310/317  (97.8%) | RR 0.58 (0.48 to 0.69) | 411 fewer per 1000 (from 303 fewer to 509 fewer) | ⊕⊕⊕O MODERATE | IMPORTANT |
|  |  |  |  |  |  |  |  | 99.1% |  | 416 fewer per 1000 (from 307 fewer to 515 fewer) |  |  |
| **Overall successful intubation - In-hospital** | | | | | | | | | | | | |
| 5 | randomised trials | no serious risk of bias | serious^2^ | no serious indirectness | no serious imprecision | none | 313/325  (96.3%) | 274/320  (85.6%) | RR 1.11 (1.01 to 1.23) | 94 more per 1000 (from 9 more to 197 more) | ⊕⊕⊕O MODERATE | IMPORTANT |
|  |  |  |  |  |  |  |  | 87.4% |  | 96 more per 1000 (from 9 more to 201 more) |  |  |
| **Duration of intubation (Better indicated by lower values)** | | | | | | | | | | | | |
| 10 | randomised trials | no serious risk of bias | very serious^1^ | no serious indirectness | very serious^3^ | none | 1084 | 1089 | - | MD 2.12 lower (13.41 lower to 9.18 higher) | ⊕OOO VERY LOW | IMPORTANT |
| **Esophageal intubation rate** | | | | | | | | | | | | |
| 6 | randomised trials | no serious risk of bias | no serious inconsistency | no serious indirectness | no serious imprecision | none | 7/628  (1.1%) | 21/617  (3.4%) | RR 0.36 (0.16 to 0.8) | 22 fewer per 1000 (from 7 fewer to 29 fewer) | ⊕⊕⊕⊕ HIGH | IMPORTANT |
|  |  |  |  |  |  |  |  | 3.8% |  | 24 fewer per 1000 (from 8 fewer to 32 fewer) |  |  |
| **Mortality** | | | | | | | | | | | | |
| 6 | randomised trials | no serious risk of bias | no serious inconsistency | no serious indirectness | serious^4^ | none | 89/741  (12%) | 80/752  (10.6%) | RR 1.12 (0.86 to 1.45) | 13 more per 1000 (from 15 fewer to 48 more) | ⊕⊕⊕O MODERATE | CRITICAL |
|  |  |  |  |  |  |  |  | 12.7% |  | 15 more per 1000 (from 18 fewer to 57 more) |  |  |
| **Aspiration rate** | | | | | | | | | | | | |
| 6 | randomised trials | no serious risk of bias | serious^2^ | no serious indirectness | serious^4^ | none | 12/788  (1.5%) | 20/800  (2.5%) | RR 1.01 (0.98 to 1.03) | 0 more per 1000 (from 0 fewer to 1 more) | ⊕⊕OO LOW | IMPORTANT |
|  |  |  |  |  |  |  |  | 1.8% |  | 0 more per 1000 (from 0 fewer to 1 more) |  |  |
| **Severe low oxygen saturation rate** | | | | | | | | | | | | |
| 4 | randomised trials | no serious risk of bias | serious^2^ | no serious indirectness | very serious^5^ | none | 29/327  (8.9%) | 24/337  (7.1%) | RR 1.43 (0.51 to 3.96) | 31 more per 1000 (from 35 fewer to 211 more) | ⊕OOO VERY LOW | IMPORTANT |
|  |  |  |  |  |  |  |  | 8.3% |  | 36 more per 1000 (from 41 fewer to 246 more) |  |  |
| **Proportion of Cormack-Lehane class 1** | | | | | | | | | | | | |
| 4 | randomised trials | no serious risk of bias | no serious inconsistency | no serious indirectness | no serious imprecision | none | 267/344  (77.6%) | 174/346  (50.3%) | RR 1.54 (1.37 to 1.74) | 272 more per 1000 (from 186 more to 372 more) | ⊕⊕⊕⊕ HIGH | IMPORTANT |
|  |  |  |  |  |  |  |  | 50.3% |  | 272 more per 1000 (from 186 more to 372 more) |  |  |

^1^ Substantial heterogeneity (P<0.1; I2>80%)
^2^ Moderate heterogeneity (P<0.1; I2<80%)
^3^ Some data are assumed normally distributed with wide confidence interval.
^4^ Few participants and few events
^5^ Few participants and few events with wide confidence interval
