# Supplementary material for: Video laryngoscopy does not improve the intubation outcomes in emergency and critical patients – a systematic review and meta-analysis of randomized controlled trials
Source: Crit Care. 2017 Nov 24;21:288. doi: 10.1186/s13054-017-1885-9 (PMC5702235; doi:10.1186/s13054-017-1885-9)
Supplement: Supplementary file 6 — VL vs. DL for first-attempt success rate. Abbreviations: VL Video laryngoscope, DL Direct laryngoscope. (DOC 35 kb) [file 13054_2017_1885_MOESM6_ESM.doc]

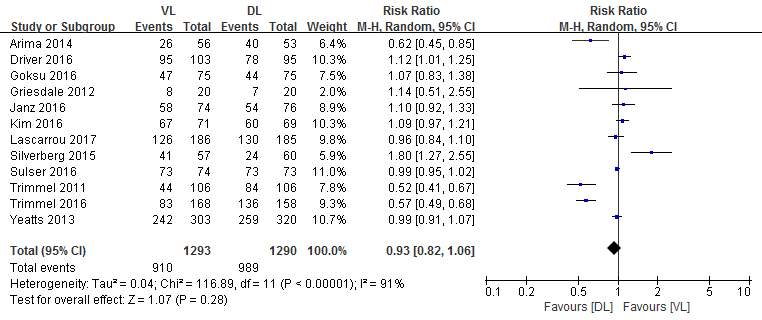


**Additional file 6: Figure S2:** VL vs. DL for first-attempt success rate. Abbreviations: VL, video laryngoscope; DL, direct laryngoscope
