# Supplementary material for: Video laryngoscopy does not improve the intubation outcomes in emergency and critical patients – a systematic review and meta-analysis of randomized controlled trials
Source: Crit Care. 2017 Nov 24;21:288. doi: 10.1186/s13054-017-1885-9 (PMC5702235; doi:10.1186/s13054-017-1885-9)
Supplement: Supplementary file 7 — Figures S3, S4, S5, and S6 VL vs. DL for overall success rate. Abbreviations: VL Video laryngoscope, DL Direct laryngoscope. (DOC 97 kb) [file 13054_2017_1885_MOESM7_ESM.doc]

**
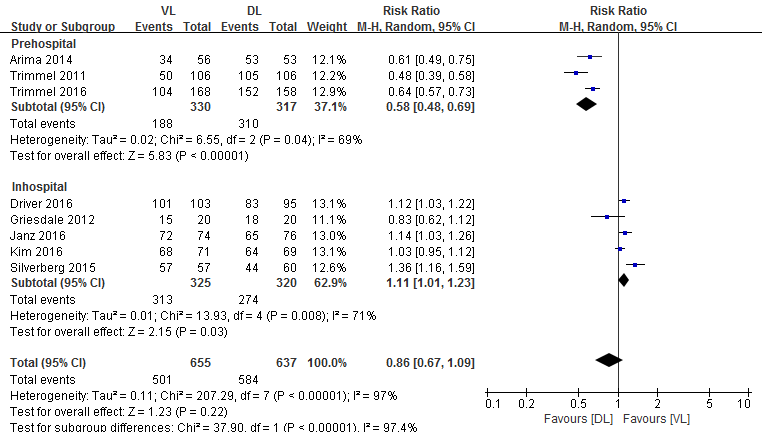
**

**Additional file 7: Figure S3:** VL *vs.* DL for overall success rate. Abbreviations: VL, video laryngoscope; DL, direct laryngoscope


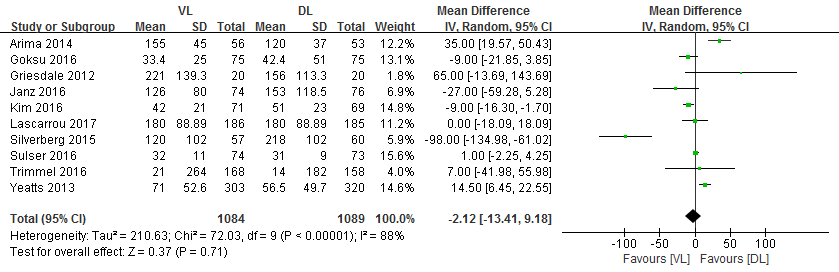


**Supplemental Digital Content-Figure 4:** VL *vs.* DL for duration of intubation. Abbreviations: VL, video laryngoscope; DL, direct laryngoscope


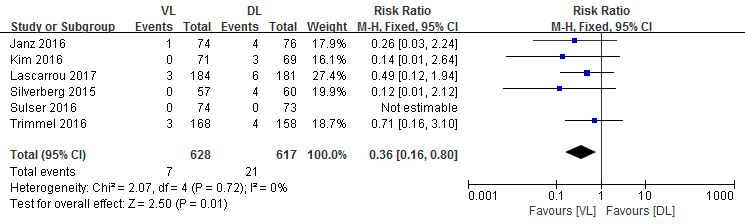


**Supplemental Digital Content-Figure 5:** VL *vs.* DL for esophageal intubation rate. Abbreviations: VL, videolaryngoscope; DL, direct laryngoscope


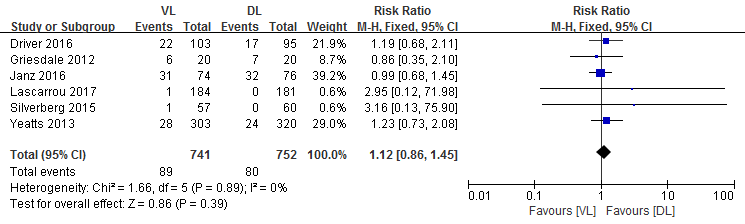


**Supplemental Digital Content-Figure 6:** VL *vs.* DL for in-hospital mortality. Abbreviations: VL, videolaryngoscope; DL, direct laryngoscope
